# The Kill Date as a Management Tool for Cover Cropping Success

**DOI:** 10.1371/journal.pone.0109587

**Published:** 2014-10-08

**Authors:** María Alonso-Ayuso, José Luis Gabriel, Miguel Quemada

**Affiliations:** School of Agriculture Engineering, Technical University of Madrid, Madrid, Spain; California State University, Fresno, United States of America

## Abstract

Integrating cover crops (CC) in rotations provides multiple ecological services, but it must be ensured that management does not increase pre-emptive competition with the subsequent crop. This experiment was conducted to study the effect of kill date on: (i) CC growth and N content; (ii) the chemical composition of residues; (iii) soil inorganic N and potentially mineralizable N; and (iv) soil water content. Treatments were fallow and a CC mixture of barley (*Hordeum vulgare* L.) and vetch (*Vicia sativa* L.) sown in October and killed on two different dates in spring. Above-ground biomass and chemical composition of CC were determined at harvest, and ground cover was monitored based on digital image analysis. Soil mineral N was determined before sowing and after killing the CC, and potentially mineralizable N was measured by aerobic incubation at the end of the experiment. Soil water content was monitored daily to a depth of 1.1 m using capacitance sensors. Under the present conditions of high N availability, delaying kill date increased barley above-ground biomass and N uptake from deep soil layers; little differences were observed in vetch. Postponing kill date increased the C/N ratio and the fiber content of plant residues. Ground cover reached >80% by the first kill date (∼1250°C days). Kill date was a means to control soil inorganic N by balancing the N retained in the residue and soil, and showed promise for mitigating N losses. The early kill date decreased the risk of water and N pre-emptive competition by reducing soil depletion, preserving rain harvested between kill dates and allowing more time for N release in spring. The soil potentially mineralizable N was enhanced by the CC and kill date delay. Therefore kill date is a crucial management variable for maximizing the CC benefits in agricultural systems.

## Introduction

The potential for cover crops (CC) to provide ecological services has been documented in diverse cover cropping systems and environments [1]. Replacing fallow periods with CC may enhance soil aggregate stability [2], water retention capacity [3], nutrient supply [4] and disease suppression [5]. Moreover, plant residues covering the soil following crop kill can improve soil protection over time and help control weeds, preserve soil moisture, ameliorate compacted soils, and reduce soil erosion [6]. However, improperly managed CC may have a detrimental effect on the cash crop either by competing for water and nutrients, building up diseases or retarding seed germination [6], [7]. Overall, there are two crucial management factors that determine CC success: species selection and CC termination [8]. In this manuscript, we focus on the termination of CC incorporated into field crop rotations, particularly on kill date. Previous experiments have shown that changing kill date by a few weeks in spring may have a large effect on the water and N pre-emptive competition with the subsequent cash crop, nitrate leaching control, and soil moisture conservation [7], [9]–[12], but there is no agreement on the advantages and limitations of postponing CC termination. Therefore, a better understanding of the effect of the living CC and its dead mulch on the dynamics of N and water might improve our ability to choose a kill date that maximizes the benefits of CC in the system.

The effect of kill date on soil water availability is a balance between the water extracted by the living CC and the evaporation prevented by the residue mulch [9]. Integrating CC into the cropping system increases above-ground biomass and resistance to decomposition, which enhances the development of a dead mulch covering the soil surface [13]. Some authors have found that, despite the soil water depletion, moisture conservation was improved when the CC was killed later [9], [11]. However, other experiments have shown that an earlier CC kill reduced water pre-emptive competition, preserved top soil moisture, and increased water availability for the subsequent crop [7], [12]. Similarly, postponing kill date enhanced N pre-emptive competition as soil available N is depleted by the CC uptake and only slowly replenished by the mineralization of a high C/N residue [6]. Legume species may compensate for this effect by fixing more N_2_ when killed late [10]. The water and N balance is driven by various factors that are all affected by kill date, such as above-ground biomass accumulation, ground cover (GC), and N uptake [6]. The chemical composition of crop residues, such as lignin content and the C/N ratio, are important characteristics governing the decomposition process and determining the persistence of the residue layer covering the soil [14]. However, there are few experiments that combine multiple approaches to study these variables, and to our knowledge, none that involves continuous monitoring of soil water dynamics.

We hypothesize that a combined study of CC growth, GC and water and N dynamics would contribute to understanding the effect of kill date and improve management practices. This experiment was conducted over two years to study the effect of kill date on the following factors: (i) the growth and N content of the CC; (ii) the chemical composition and residue quality of the CC; (iii) soil inorganic N dynamics and its potentially mineralizable N; and (iv) the soil water content. A CC mixture of barley and vetch was selected because mixing legumes and non-legumes is known to be an efficient technique to merge N and water management benefits of the individual species [9], [15].

## Materials and Methods

### 1. Field experiment

**Figure 1:**
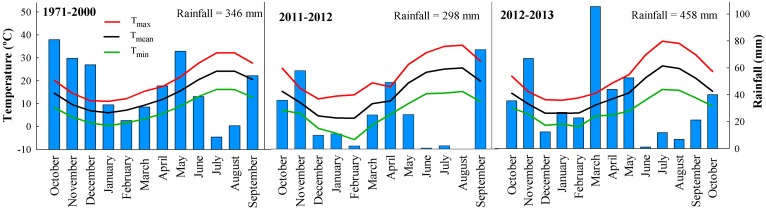
Monthly rainfall (bars) and average maximum, mean and minimum temperatures from 1971 to 2000 and during the two experimental seasons at Aranjuez (Madrid, Spain).

The experiment was conducted from October 2011 to November 2013 at La Chimenea field station (40°03′N, 03°31′W, 550 m a.s.l.), which is located in the central Taxus river basin in Aranjuez (Madrid, Spain). The soil is a silty clay loam (Typic Calcixerept) [16] with a high content of organic matter and carbonates, pH ∼8 and low stone content throughout the profile. The most relevant soil characteristics are presented in Table 1. Total organic C was measured by the Walkley-Black method [17]. Calcium carbonate was determined by a volumetric calcimeter method [18]. The climate is semiarid Mediterranean [19] with high interannual variability (Figure 1). Annual rainfall averages ∼350 mm with less rain in the summer and more in the autumn and a mean annual temperature of 14.2°C. Weather data were recorded throughout the experimental period by a data logger (CR10X. Campbell Scientific Ltd, Shepshed, UK) located ∼100 m from the field site.

**Table 1:** Soil properties at the beginning of the experiment.

Depth (cm)	0–23	23–40	40–70	70–120
pH (1∶2.5)	8.16	8.06	8.02	7.84
Organic Matter (g kg^−1^)	31.8	29.2	21.9	22.3
CO_3_ (g CO_3_^2−^ kg^−1^)	198.0	201.3	159.0	181.0
Sand (g kg^−1^)	260	250	250	250
Silt (g kg^−1^)	490	510	520	460
Clay (g kg^−1^)	250	240	230	290

The experiment consisted of twelve plots (15 m×12 m) randomly assigned to three treatments (fallow and two different CC kill dates) with four replications. Cover crop treatments were sown with a barley (*Hordeum vulgare* L., cv. Vanessa) + vetch (*Vicia sativa* L., cv. Aitana) mixture in early October (October 6, 2011; October 8, 2012). The mixture, 30% (54 kg ha^−1^) barley seeds and 70% (45 kg ha^−1^) vetch, was broadcast by hand and buried (∼5 cm) with a shallow cultivator. All treatments received ∼18 mm irrigation on the sowing date with a sprinkle irrigation system (9.5 mm h^−1^) to ensure uniform CC establishment. Plots from the first kill treatment (FK) were killed in mid-March (March 13, 2012; March 14, 2013) when barley was at the end of the booting growth stage and vetch at the stem elongation stage. Plots from the second kill treatment (SK) were killed in mid-April (April 9, 2012; April 13, 2013) when barley was at the emerging inflorescence growth stage and vetch was at the stem elongation stage. Cover crops were killed by application of glyphosate (N-phosphonomethyl glycine, 0.7 kg a.e. ha^−1^) followed by a shredder. The last day of April was chosen as a hypothetical planting date (HPD) for both years, as it is a typical date for planting summer cash crops in the area. The HPD represented the beginning of the mulch period during which the CC residue remained on the ground. To compare the residue mulch effect on water conservation, SWC was homogenized by adding water to the drier plots with a drip irrigation system (30.5 mm to FK treatment and 35.5 mm to SK) in April 2012. As rainfall was abundant in March 2013, no supplemental irrigation was applied. Fallow plots were kept free of weeds by hand weeding during the trial period. The experiment was conducted in a field that was cultivated with triticale during the two previous years and had not received organic amendments or N fertilizer for four years prior to the beginning of the trial.

### 2. Cover crop analysis

To measure the above-ground biomass and GC in each plot, four 0.5 m×0.5 m squares were marked after sowing the CC. Ground cover was monitored in each square based on digital photos taken from a nadir perspective at 1.2 m height every other week [20]. The images were taken with a Nikon S210 Coolpix camera that had a lens resolution of 5 Mpixels. The images were saved at a resolution of 1200×800 pixels and the GC percentage was determined with the software SigmaScan Pro (Systat software, Chicago, IL, USA) by means of the “Turf analysis” macro developed by Karcher and Richardson [21]. The process consisted of creating a pixel layer by selecting the hue and saturation ranges that identified the surface covered by the crop. The GC was determined to be the number of pixels of the layer divided by the total number of pixels constituting the image of the marked squares [20]. The GC evolution was adjusted to the Gompertz function [22], and the thermal times (°C days) until the GC reached 30% (t_30_) and 80% (t_80_) were calculated.

Just before killing the CC, the above-ground biomass of the marked squares was hand harvested by cordless grass shear at the ground level, separated by species, oven dried for 48 h at 65°C, and weighed to determine biomass of barley, vetch, and mixture in kg ha^−1^. The C and N concentrations in the above-ground biomass were determined for a subsample of each species from each plot using the Dumas combustion method (LECO CHNS-932 Analyzer, St. Joseph, MI, USA). Total N content was calculated for each specie as the product of above-ground biomass times N concentration. The atmospheric N_2_ fixation by the legume was estimated by the natural abundance method [23] based on the δ^15^N (‰) determination (Europe Scientific 20–20 IRMS Analyzer, Crewe, UK) for subsamples from the vetch and a barley reference cultivated as a sole crop in an adjacent field. Cover crop residue quality was assessed by measuring neutral detergent fiber (NDF), acid detergent fiber (ADF), and lignin (L) with the Goering and Van Soest method [24] in subsamples of each crop species from each plot.

### 3. Soil inorganic nitrogen content (N_min_)

Four soil cores were taken from each plot to a depth of 1.2 m in 0.2 m intervals with an Eijkelkamp helicoidal auger (Eijkelkamp Agrisearch Equipment, Geisbeek, Netherlands) just before sowing the CC, after the second kill date each year, and at the end of the experiment. Soil cores were combined by depth to provide a composite profile of six samples. For each plot, soil N_min_ was calculated for each layer. Soil samples were placed in a plastic box and immediately firmly closed then transported, and refrigerated (4–6°C). Within five consecutive days, a soil subsample from each box was extracted with 1 M KCl (∼30 g of soil: 150 ml of KCl), centrifuged, decanted, and a subsample of the supernatant volume was stored in a freezer until later analysis. Nitrate concentration in the extracts was determined by the Griess-Ilosvay method [25] after reduction of NO_3_^−^ to NO_2_^−^ with a Cd column. Ammonium in the soil extracts was determined by the salicylate-hypochlorite method [26].

### 4. Soil nitrogen mineralization potential (N_0_)

Soil N mineralization potential was estimated by adapting the procedure proposed by Stanford and Smith [27]. A subsample of the top soil layer (0–0.2 m) free from residue contamination was collected from each plot after the second kill date of the second year, air-dried, and sieved (<6 mm). A homogeneous mixture of 30 g of that soil and an equal weight of sand was packed to a depth of 5 cm in plastic syringes (3.5 cm diameter, 10 cm long) to achieve a bulk density of 1.25 g cm^−3^. Thin glass wool layers were placed between the soil and the bottom of the syringe and over the soil sample to avoid soil loss during leaching and to minimize moisture loss. Initial soil inorganic N was removed by leaching with 100 ml of 0.01 M CaCl_2_ followed by 20 ml of a N-free nutrient solution (0.0095 M CaSO_4_, 0.000047 M KH_2_PO_4_, 0.00138 M K_2_SO_4_, 0.0003 M MgSO_4_). The excess water was removed using a vacuum pump until a weight within 2 g of that measured before leaching was reached. The syringes were covered with a porous parafilm and incubated aerobically at 35°C. Syringes were removed from the incubator at 14, 28, 42, 56, and 60 d after preparation and were leached with 100 ml of 0.01 M CaCl_2_ solution followed by 20 ml of N-free solution. Leachates were made up to 100 ml with CaCl_2_, and subsamples were stored in a freezer at −25°C until later analysis. After the leaching procedure, the cores were allowed to drain under vacuum until a weight within 2 g of that measured before leaching was reached. Nitrate concentrations in the leachates were determined by the Griess-Ilosvay method, ammonium by the salicylate-hypochlorite method and total N by the Dumas combustion method, as described above. The N_0_ and the mineralization rate constant (k) were estimated after fitting a non-linear regression model (Nt = N_0_ exp (−k t)) for describing the cumulative N mineralized (Nt) with time (t) in each soil sample.

### 5. Soil volumetric water content (SWC)

The SWC was monitored daily during the field trial using the EnviroScan capacitance probes (Sentek Pty Ltd, Stepney, Australia) that has been described in detail elsewhere [28]. Seventy-two capacitance sensors were mounted on twelve plastic extrusions (four repetitions per treatment), introduced into access tubes located in the middle of each plot and connected to three data loggers. Sensor readings were automated and stored in the data loggers and downloaded weekly. To ensure the measurement reliability, a normalization procedure was conducted that obtained reference readings by exposing each sensor to air and water (∼20°C). The sensors were centered at 10, 30, 50, 70, 90, and 110 cm below the soil surface in each plastic extrusion, and normalized readings were registered every 6 h. A daily average of the four readings from the 0–20 (10), 20–40 (30), 40–60 (50), 60–80 (70), 80–100 (90), 100–120 (110) cm-deep soil layers was transformed into SWC using a calibration equation that was obtained at the experimental site [29]. The SWC data set was comprised of two CC seasons, which started before sowing in October and lasted until August each year, and used to study the effect of the living CC and the dead mulch on soil moisture.

### 6. Statistical analysis

Analyses of variance (ANOVA) and t-test were performed in order to determine differences between kill dates for each variable. Year was considered a random effect and, interaction between kill date and year was also tested. Means were separated by Tukey’s test at the 0.05 probability level (P≤0.05). Least significant differences (LSD) were calculated for SWC (P≤0.05). Prior to conducting the ANOVA, tests were conducted to verify if the assumptions of ANOVA were met. The Gompertz model was fitted to the GC and the N mineralization potential model was fitted to the cumulative N mineralized using a non-linear regression procedure. The models were evaluated for their ability to simulate the observed data by comparing the mean of the lack of fit to the mean square due to pure error by using the variance ratio, or F-test. When the lack of fit was significantly smaller than the pure error, the model fitted the data. Further discussion regarding this evaluation procedure can be found elsewhere [30], [31]. Statistical analyses were accomplished using the PASW Statistics Software (SPSS, Chicago, IL, USA).

## Results

### 1. Weather conditions

The first experimental year was drier than the second (Figure 1). In the first year, rainfall was substantially lower (298 mm) than the 30-year average although in the second, it was greater (408 mm). Differences mainly occurred in spring; from March to May, the rainfall was 202 mm in 2013 compared with 101 mm in 2012. The rest of the season was rather similar for both years except for intense precipitation in late September 2012. Air temperature followed a typical Mediterranean seasonal distribution over the two years. Winter was cooler in the first year with an average minimum temperature of −3 and −5°C in January and February, respectively.

### 2. Cover crop: GC, above-ground biomass and N content

**Figure 2:**
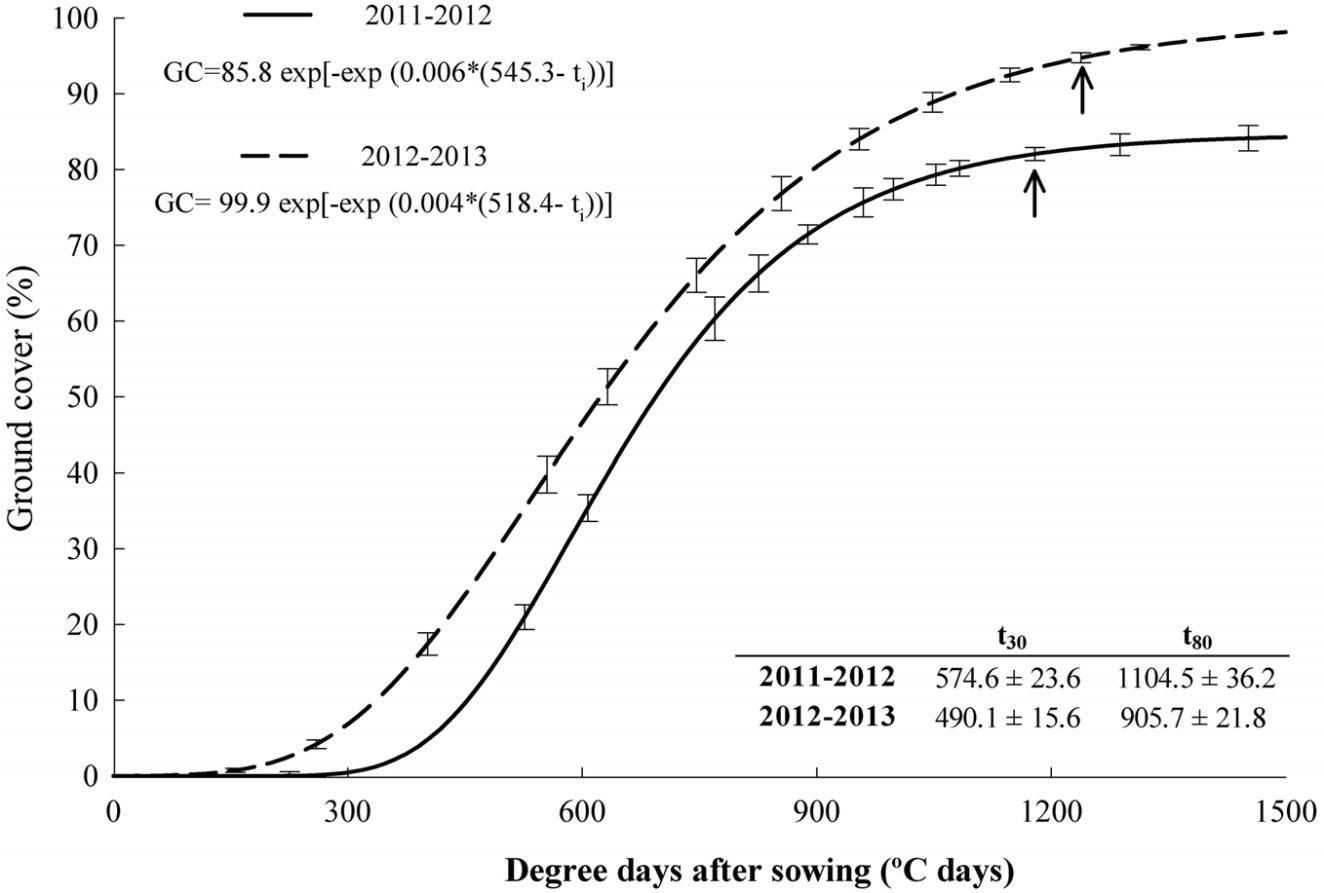
Ground cover (GC) development during the cover crop growth period in both experimental seasons. Arrows show the first kill date. Lines represent the Gompertz model adjusted to the observed values. Fitted models and the thermal time until the ground cover reaches 30 and 80% (t_30_ and t_80_) are shown.

**Figure 3:**
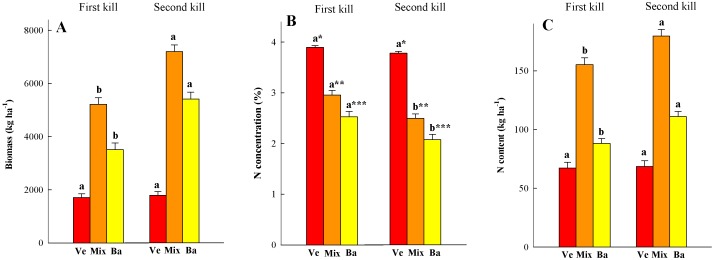
Biomass (A), N concentration (B) and N content (C) for vetch (Ve), barley (Ba) and the mixture (Mix) measured for the first and second kill dates. Values are the mean of two cropping seasons. Letters above bars show significant differences between kill dates for each species, and asterisks between species for each kill date. Small bars represent the standard error.

The GC followed a classical Gompertz model (Figure 2). The F-test comparing the mean squares due to pure error and lack of fit was not significant at 0.01 level, therefore, the model was adjusted to the GC observations. The first kill occurred between 157 and 159 d after sowing (∼1200°C days) whereas the second year occurred between 184 and 186 d after sowing (∼1500°C days). The GC>30%, a crucial threshold for erosion control [32], was attained between 37 and 41 d after sowing (>490°C days). The GC>80%, considered to be full cover for erosion control and direct soil evaporation [32], was reached between 95 and 130 d after sowing (>900°C days). Differences were observed in the maximum GC attained, 85% in 2012 and 100% in 2013. In both years, maximum GC was already attained by the first kill date. The ground continued to be covered by the CC residue mulch more than six months after CC killing. Above-ground biomass increased from the FK to the SK by ∼2000 kg ha^−1^, and most of the increase was due to barley (Figure 3). Although barley biomass was greater at the SK than at the FK, no differences were found in vetch biomass between kill dates. Vetch above-ground biomass was double in 2013 than in 2012 whereas barley above-ground biomass did not vary by year. At the end of the mulch period, residues left on the soil surface were greater in the SK (2214 kg ha^−1^ in 2012, and 4014 in 2013) than in the FK treatment (966 kg ha^−1^ in 2012, 2151 in 2013. The ground was fully covered by the residues in both treatments.

Nitrogen concentration in the above-ground biomass was higher in the FK than in the SK. Nitrogen concentration in the mixture decreased from 2.7 to 2.35% in 2012 and from 3.2 to 2.6% in 2013. As expected, vetch had a higher N concentration than barley whereas the N concentration of the mixture was intermediate (Figure 3). Differences in N content between the FK and SK, calculated as the product of above-ground biomass and N concentration, were not significant for the mixture or vetch (Figure 3). However, barley N content in the SK was higher than in the FK; N uptake increased from 79 to 107 kg N ha^−1^ in 2012 and from 97 to 115 kg N ha^−1^ in 2013. The vetch reached high N_2_ atmospheric fixation rates during the experiment. In the first season, N_2_ atmospheric fixation was >80% of N content and ∼100% in the second. No differences in N_2_ atmospheric fixation were found between kill dates.

**Figure 4:**
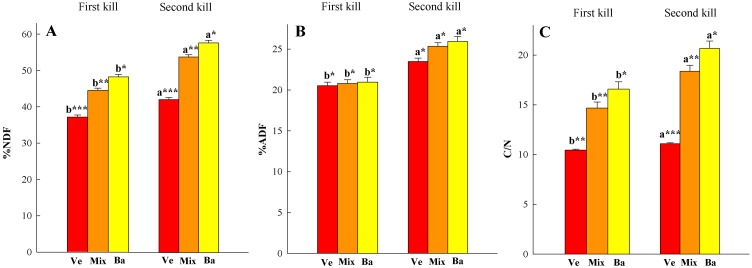
Neutral detergent fiber-NDF- (A) and acid detergent fiber -ADF- (B) fractions, and C/N ratio (C) in vetch (Ve), barley (Ba) and the mixture (Mix), measured for the first and second kill dates. Values are the mean of two cropping seasons. Letters above bars show significant differences between kill dates for each species, and asterisks between species for each kill date. Small bars represent the standard error.

Residue quality fractions varied between kill dates (Figure 4). The NDF and ADF fractions increased when the CC was killed later. An interaction effect between treatment and year occurred for lignin content as differences in the lignin fraction between kill dates were only significant in barley and in the mixture in 2012. Lignin fraction in the mixture increased from 2.2 to 3.6% and in the barley from 1.2 to 2.9% in 2012. The C/N of barley, vetch and mixture residues was always lower for the FK than for the SK.

### 3. Soil inorganic nitrogen content

**Figure 5:**
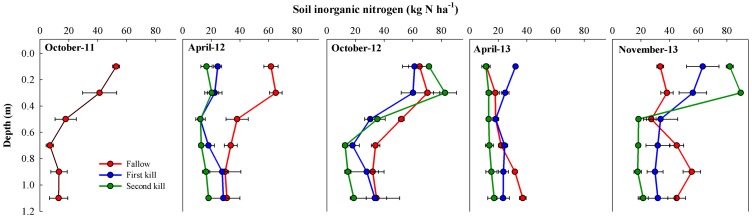
Soil inorganic nitrogen (kg N ha^−1^) in the upper 1.2 m of the soil profile for the fallow (Fa), first kill (FK) and second kill (SK) treatments at different sampling times.

At the beginning of the experiment, average N_min_ of the upper layers of the soil profile was 145 kg N ha^−1^ (Figure 5, Table 2). During the first CC growing period, soil N_min_ increased in the fallow treatment, particularly in the upper 0.8 m, while it decreased in the remaining depths. During the mulch period, soil N_min_ in the fallow treatment increased slightly whereas, in the CC treatments, N_min_ increased largely in the upper soil layers, but there was no difference between the treatments by October in 2012.

**Table 2:** Soil inorganic N content (kg N ha**^−^** ^1^) in the different soil layers and the entire profile at different sampling dates for the fallow (Fa), first kill (FK) and second kill (SK) treatments.

	Oct 2011	Apr 2012	Oct 2012	Apr 2013	Oct 2013
**0–40 cm**					
Fa	94 (13.7)	126.8 (7.9) a	135.3 (15.4)	29.7 (3.8) b	71.54 (5.38) c
FK	94 (13.7)	46.9 (7.1) b	121.5 (16.6)	56.9 (3) a	119.37 (28.48) b
SK	94 (13.7)	37 (8.2) b	154 (8)	24.8 (4.5) b	171.72 (2.36) a
**40–80 cm**					
Fa	24.7 (9.5)	71.6 (10.6) a	86.1 (4.6) a	40.1 (1.8) a	72.22 (7.43)
FK	24.7 (9.5)	30.4 (5.3) b	48.3 (4.4) b	42.6 (3.5) a	65.38 (19.89)
SK	24.7 (9.5)	25.4 (5.1) b	48.1 (7.1) b	27.1 (4.1) b	39.39 (2.68)
**80–120 cm**					
Fa	26.1 (11.1)	60.6 (3.5)	66.7 (11)	68.8 (3.3) a	100.37 (11.74) a
FK	26.1 (11.1)	56.1 (24.5)	61.5 (29.3)	47 (7.4) b	61.59 (17.91) ab
SK	26.1 (11.1)	34.5 (3.7)	33.5 (6.3)	32.3 (8.3) b	39.24 (5.83) b
**Soil profile (0–120 cm)**					
Fa	144.8 (22.8)	259 (8.0) a	288.1 (25.4)	138.6 (5.1) a	244.13 (19.05)
FK	144.8 (22.8)	133.32 (27.5) b	231.3 (31.2)	146.6 (10.7) a	246.34 (52.87)
SK	144.8 (22.8)	96.9 (15.4) b	235.6 (14.2)	84.2 (13.3) b	247.34 (7.9)

Means with standard error in parentheses.

Within a date and depth, means with the same letter are not significantly different between kill dates at P<0.05.

During the second CC growing season, soil N_min_ was depleted in all treatments. After the CC kill in April 2013, the N_min_ was 84 kg N ha^−1^ in the SK treatment and ∼140 kg N ha^−1^ in the others. The FK treatment accumulated more N_min_ in the upper layers, most likely due to the early mineralization that occurred between the CC kill and soil sampling (∼4 weeks). At the end of the mulch period in November 2013, a large increase in soil N_min_ occurred in all treatments. Although no difference was observed for average N_min_ (∼245 kg N ha^−1^) over the entire profile, differences between treatments were observed at some soil depths. In the SK, most of the N_min_ (∼70%) was in the top layer (0–40 cm) whereas it was ∼29% in the fallow treatment. Similarly, while >40% of soil N_min_ was in the bottom layer in the fallow, <16% was in the bottom layer in the SK. The N_min_ distribution in the FK treatment was intermediate between the others.

### 4. Soil nitrogen mineralization potential

**Figure 6:**
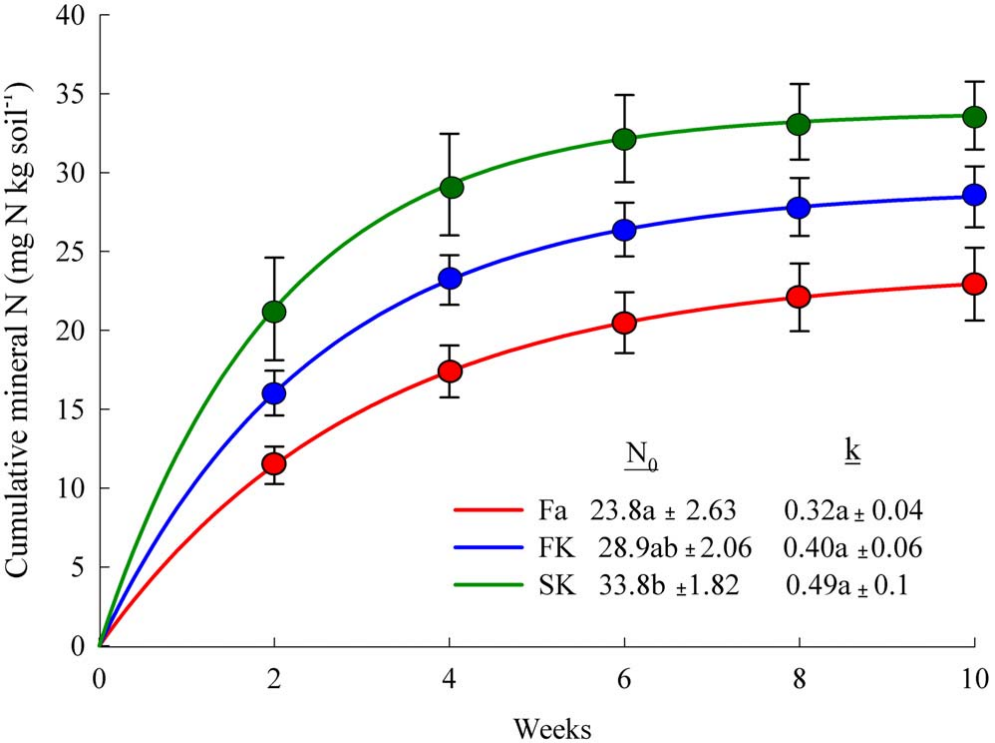
Cumulative N mineralization in soils from the fallow (Fa), first kill (FK) and second kill (SK) treatments during a 10-week aerobic incubation. Soil N mineralization potential (N_0_) and N mineralization rate (k) were calculated by fitting a non-linear regression model (Nt = N_0_ exp (-k t)). Values followed by different letters are significantly different between treatments at ≤0.05 by Tukey’s test. Bars represent the standard error.

The one-pool exponential model fit the cumulative mineral N from the aerobic incubation, as the F-test comparing the mean squares due to pure error and lack of fit was not significant at 0.01 level. The N_0_ was higher for soils from the SK treatment (34 mg N kg soil^−1^) than those from the fallow (24 mg N kg soil^−1^). The N_0_ from the FK treatment was intermediate. No differences between treatments were observed in the N mineralization (Figure 6).

### 5. Soil water content

**Figure 7:**
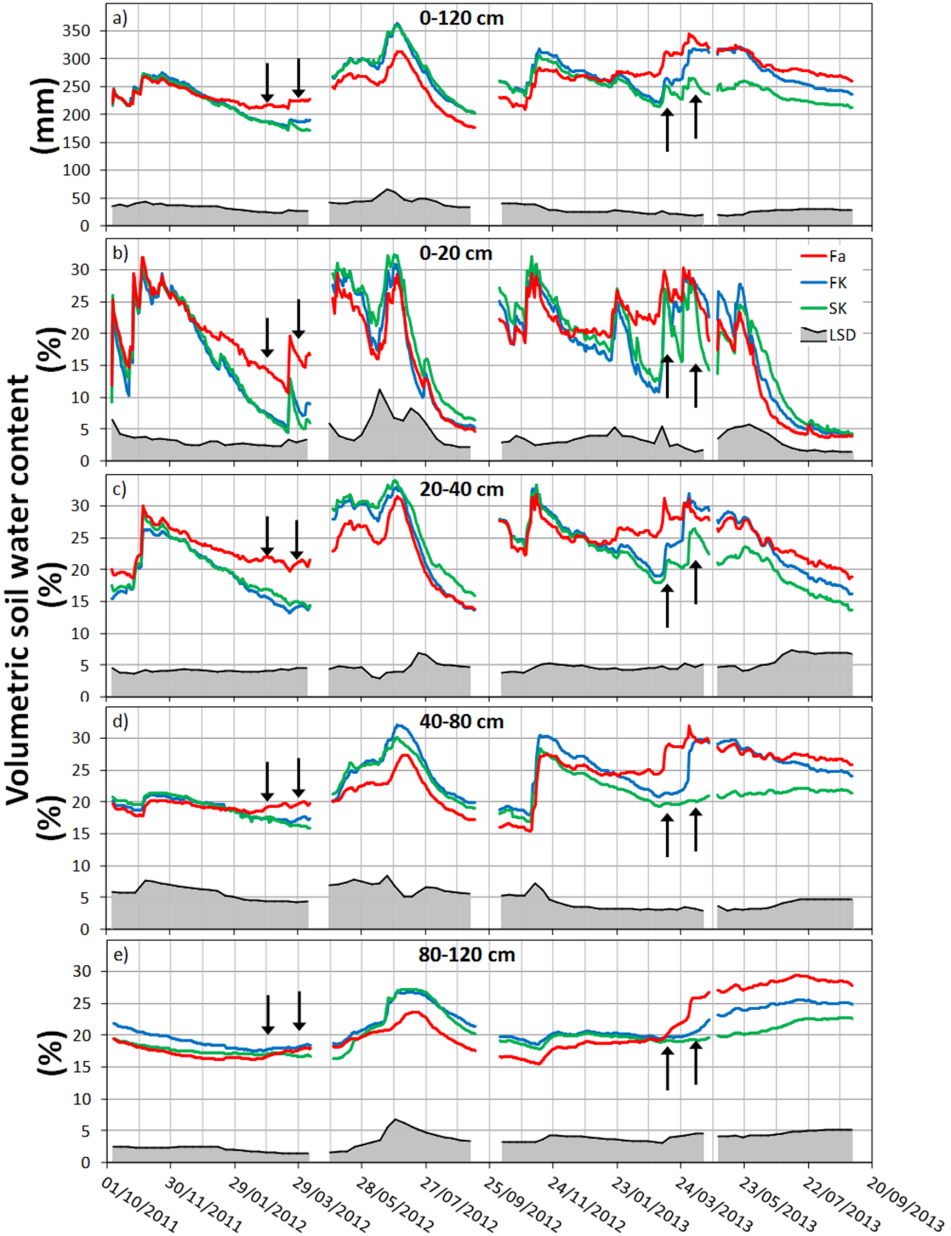
Soil water content monitored with the capacitance sensors at different depths for the fallow (Fa), first kill (FK) and second kill (SK) treatments during the experimental period. Arrows represent the first and second kill dates. The shadowed area in the bottom represents the LSD at 0.05 probability level.

Soil water content was affected by the presence of CC and by the kill date with differences observed in the upper layers and the whole soil profile (Figure 7, Table 3). During both CC growth periods, SWC followed a similar pattern. At sowing, the three treatments started with low SWC in the entire profile (∼220 mm) in both seasons, and only small differences appeared in the top layers. The precipitation during the three months following CC sowing recharged the soil profile, reaching SWC>300 mm in 2013 due to abundant rainfall (Figure 7a). No differences were observed between treatments during this period in any season. However, during the next three months, CC extracted water from the upper layers, and by the first kill date, SWC was higher in the fallow than in the CC treatments. After the first kill date, the SWC in the FK treatment varied compared to the SK depending on the annual weather conditions. During the first season, no differences were observed between the FK and SK treatments, most likely due to low precipitation, and by the second kill date, both had similar SWC and were lower than the fallow (Table 3). During the second season, the high precipitation between both kill dates recharged the soil profile, and the FK treatment SWC was similar to the fallow except in the deeper soil layer (100–120 cm, Figure 7e) where the fallow treatment remained wetter. For the second kill date of 2013, the SK treatment SWC was lower, and differences with the FK were obvious down to 80 cm and down to 120 cm with the fallow (Table 3).

**Table 3:** Soil water content (mm) in each of the soil layers and the entire profile at different sampling dates for the fallow (Fa), first kill (FK) and second kill (SK) treatments.

		0–20		20–40		40–60		60–80		80–100		100–120		0–120	
		-------------------------------------------------------------------------------------------------------------mm----------------------------------------------------------------------------------------------------													
**2011–2012**															
07/10/2011	Fa	23.9	a	40.3	a	38.1	a	40.8	a	37.4	a	40.4	a	221	a
**CC sowing**	FK	18.7	a	30.8	b	39.4	a	41.7	a	44.2	a	43.4	a	218	a
	SK	18.5	a	35.1	ab	42.6	a	41.0	a	37.0	a	41.0	a	215	a
13/03/2012	Fa	25.2	a	42.7	a	40.5	a	37.5	a	34.1	a	34.9	a	215	a
**First kill date**	FK	12.4	b	28.7	b	33.3	a	35.5	a	35.5	a	36.5	a	182	b
	SK	11.4	b	31.7	b	35.5	a	32.2	a	33.1	a	35.5	a	179	b
09/04/2012	Fa	33.9	a	42.2	a	40.1	a	38.3	a	35.4	ab	36.3	a	226	a
**Second kill date**	FK	18.3	b	28.1	b	33.2	a	36.0	a	36.3	a	37.5	a	189	b
	SK	12.6	c	28.6	b	33.8	a	29.8	a	30.8	b	36.4	a	172	c
30/04/2012	Fa	9.4	c	27.6	c	32.6	a	36.2	a	34.1	ab	36.4	a	251	a
**HPD**	FK	10.6	b	27.4	b	36.9	a	42.7	a	41.5	a	44.0	a	266	a
	SK	12.8	a	32.0	a	38.7	a	37.3	a	37.4	b	43.6	a	269	a
12/09/2012	Fa	9.4	a	27.6	a	32.6	a	36.2	a	34.1	b	36.4	a	176	a
**Mulch period end**	FK	10.6	a	27.4	a	36.9	a	42.7	a	41.5	a	44.0	a	203	a
	SK	12.8	a	32.0	a	38.7	a	37.3	a	37.4	ab	43.6	a	202	a
**2012–2013**															
08/10/2012	Fa	43.9	b	55.3	a	31.3	a	33.6	a	32.0	b	34.4	a	231	a
**CC sowing**	FK	48.5	ab	55.7	a	36.3	a	39.7	a	38.5	a	40.5	a	259	a
	SK	52.7	a	55.2	a	39.0	a	34.7	a	35.3	ab	41.0	a	258	a
15/03/2013	Fa	50.3	a	56.0	a	58.8	a	56.4	a	44.7	a	39.1	a	305	a
**First kill date**	FK	43.4	b	47.3	b	41.1	b	43.8	b	40.8	a	38.3	a	255	b
	SK	41.2	b	42.2	c	39.3	b	39.3	b	37.4	a	39.2	a	239	b
11/04/2013	Fa	49.4	b	56.1	a	57.7	a	60.0	a	55.9	a	47.7	a	327	a
**Second kill date**	FK	53.9	a	59.1	a	61.2	a	58.2	a	43.8	b	39.8	b	316	a
	SK	39.1	c	50.4	b	41.0	b	40.1	b	37.8	b	39.3	b	248	b
30/04/2013	Fa	44.2	b	52.6	a	54.6	a	57.6	a	55.6	a	51.8	a	311	a
**HPD**	FK	52.7	a	55.4	a	57.6	a	59.5	a	50.0	a	42.4	b	307	a
	SK	40.3	c	41.7	b	42.2	b	40.8	b	38.8	b	40.5	b	244	b
01/09/2013	Fa	7.8	a	37.7	a	47.0	a	56.2	a	56.5	a	54.9	a	315	a
**Mulch period end**	FK	8.5	a	32.5	ab	45.3	a	50.9	a	50.1	ab	49.2	a	318	a
	SK	8.8	a	27.5	b	41.4	a	44.0	a	43.3	b	47.2	a	244	b

Within a date and depth, means with the same letter are not significantly different at P<0.05.

Although initial differences in SWC were observed between the two years of the study during the mulch period, such differences were not evident toward the end of the season. After water application in April 2012, the SWC in the entire profile at the HPD was similar in all treatments (∼262 mm; Table 3). During the wet/dry cycles that occurred in spring and summer, the CC treatments always retained more moisture than the fallow which showed the ability of the residue mulch to reduce soil water evaporation losses (Figure 7). After mid-June, when the soil was left to dry under the summer heat, differences in SWC between treatments disappeared, first in the upper layers and later in the entire soil profile. Even though slower drying was observed in the CC treatments, no differences were observed between treatments by the end of September (Table 3). In 2013, no water was applied, and the SWC in the entire soil profile was lower for the SK than for the FK and fallow treatments at the HPD (Table 3). At this time, no differences in SWC were observed between the FK and fallow treatments in the entire soil profile. However, the FK upper layer was wetter than the fallow because of the reduction in soil water evaporation caused by the residue mulch (Table 3, Figure 7b). During summer, water losses in the top layer were greater in the fallow than the CC treatments, showing the effect of the mulch. By the end of the experiment, no differences in the SWC of the entire profile were observed between treatments, but some SK subsoil layers remained drier as the result of water depletion caused by the living CC.

## Discussion

This study confirmed that CC kill date is an important management strategy as it affected key variables of the soil-plant system, influencing the success of the subsequent cash crop. Previous studies have focused mainly on the effect of N on the succeeding crop [6] and on the CC residue quality [14], but there is a lack of information on the combined effects of CC growth, N dynamics, and water dynamics. This information is necessary to optimize CC benefits and for development of more accurate simulation models.

Delaying kill date did not increase GC whereas the year had an effect on the maximum cover attained. We could not find data to compare the effect of kill date on GC. Nevertheless, our results agreed with those of previous researchers that showed the capacity of the asymptotic Gompertz function to capture the characteristics of the GC evolution of several CC [22], [33]. Ground cover is an important variable used in studies related to soil erosion [32], evaporation [34], weed control [35], [36] or radiation interception [22]. In addition, the evolution of digital technologies allows for non-destructive monitoring using reliable and efficient techniques [20]. For all of these reasons, we propose that GC should become a common variable for the characterization of CC that facilitates comparisons between species, varieties and management strategies in different regions. In the present study, GC differed with seasonal weather conditions; the dry 2011–2012 season was less favorable for CC growth than the wet and mild 2012–2013 season. In both seasons, GC>80% was already attained by the first kill date, so no additional benefits in terms of soil erosion or weed control were expected by the second kill date [32], [35].

As expected, delaying kill date increased the above-ground biomass of the barley/vetch mixture. This increase was mainly due to barley as there was no difference in vetch biomass between kill dates. The barley/vetch ratio, expressed as seed weight at sowing, was 3∶7, but barley was a stronger competitor due to faster establishment and growth rate [20]. The observed increase in biomass is common in many other studies [8], [13], but the relationship between the legume and the non-legume differed. In Central Italy, a barley/vetch mixture with a 1∶1 sowing rate yielded between 48 and 66% vetch biomass 160 d after planting which increased vetch in the sowing mixture up to 75% and produced between 53 and 76% vetch biomass at harvest [37]. A possible explanation for the large barley dominance in our study is that soil available N was not a limiting factor. Other authors reported similar superior dry matter values for the non-legume compared with the legume in mixtures, and this dominance became stronger with higher levels of N [38]. This hypothesis is corroborated by the plant and soil available N results in our study. Most of the N content in the vetch/barley mixture accumulated before the FK date. Nitrogen content of barley was greater at SK than FK (Figure 3), and in the SK treatment, the inorganic N in the soil was depleted in April, particularly from the deeper layers (Table 2, Figure 5). The high competition with barley for soil N forced the vetch to rely mainly on N_2_ fixation for its N requirement, which explains the large degree of N_2_ fixation observed in the legume. We were expecting the vetch to fix a significant amount of N_2_ between both kill days, as observed in other experiments [10], [13], but no increase in vetch N content was observed as in the study of Benincasa et al. [8]. The strong competition with barley for light explains the low vetch growth between kill dates and reinforces the need for sowing mixtures with a large proportion of vetch seeds (>50%) to favor legume activity. Most authors have found that the N content in CC and mixtures increased as kill date was delayed [10], [13], but our results show that the main effect of delaying kill date increased above-ground biomass rather than N content.

The effect of postponing kill date on chemical residue composition was in agreement with previous findings in the literature [8], [12], [39]. Nitrogen concentration decreased from the first to the second kill date, being greater for vetch than for barley (Figure 3). These differences were reinforced in the C/N ratio as a slight increase in C content was observed in the SK samples compared with the FK (Figure 4). Vetch N concentration was similar to that found by other authors, but barley values were greater than those reported in other CC experiments [10], [40], [41]. In the FK samples, barley N concentration was ∼2.9% and ∼2.2% in the SK which agrees with data from barley cultivated as a cash crop using synthetic N fertilizer [42]. These data confirm that barley did not suffer from lack of N. The increase in fiber fractions, which is related to a decrease in the labile fraction, from delaying kill date was consistent with other studies and was expected to lead to slower C and N mineralization [43]. Increases in lignin fraction were only significant in barley in the first season, but this is not surprising as there were only three weeks between kill dates, and lignin content is less sensitive to change [39]. In the drier season (2011–2012), barley residue and the mixture had significantly lower fiber and lignin content than in the wetter season for both kill dates. A reduction in NDF, ADF and lignin cereal crop straw in dry years was previously noted in a study conducted over several years and locations in Washington State [44]. Our results confirmed that delaying kill date increased the amount of CC residue and the residues were more recalcitrant to decomposition. This is particularly interesting when the goal is preserving soil moisture [11], controlling weeds [35], or increasing the slow release of the N pool [45]. In the present experiment, a detailed study of CC residue decomposition was not conducted primarily because the lowest part of the residue layer stuck to the soil when sampling, and we decided that it was reliable for determining the amount of the remaining residue but not its quality.

The topsoil N_0_ was also affected by the kill date two years after starting the experiment. Soil N mineralization potential assess the capacity of a soil to mineralize N and is a good indicator of land use as it is very sensitive to small changes in labile C and N fractions [46]. The increase of N_0_ in the CC treatments proves that CC contribute to the enhancement of labile soil organic pools. The low contribution of CC to total organic C is a known fact as residues contain low quantities of recalcitrant organic matter [2]. However, CC add substrate for soil microorganisms during their growth period (root exudation, root turnover), and residue decomposition increases the organic matter labile fractions [6]. Many studies of N mineralization from CC residues have been published, but there is a lack of studies that focus on the effects on the soil in which they were cropped. In our study, no differences were observed in total soil C, but delaying kill date increased the C supplied by CC and, as a consequence, topsoil N_0_. Nitrogen mineralization rate, a parameter that mainly depends on environmental conditions, was not different between treatments and was within the range found in the literature [47].

Consistent with other studies, CC absorbed most of the available soil inorganic N during their growth period, modifying the degree of pre-emptive competition and the risk of N loss with respect to the fallow (Table 2, Figure 5). This study also showed that kill date affected these parameters and could be a means to adapt soil N availability to the requirements of a particular agroecosystem. Delaying kill date increased the risk of pre-emptive competition, a negative effect that was reflected not only in the deeper layers as is common [4], [48], but in the upper layers during the second season as well. The early kill date allowed more time for N release from CC residues, and by the HPD, more N was available in the topsoil. This positive effect of an early kill date on reducing pre-emptive competition has not been shown before and was evident when comparing FK with the SK and the fallow in the wet year, in which N_min_ was washed out of the soil during the fall and winter. The well-known CC effect of recycling N in the soil-plant system by uptaking inorganic N from deeper layers and subsequently releasing it to the top layers was clear in this study [6]. In the first season, the soil N_min_ depleted in April by the CC uptake was replenished by October by the N released from the residue mineralization. In the second season, CC prevented most of the available N from being lost from the soil, as happened in the fallow, and released the N by November. The difference in November was not in the amount of N_min_ but its distribution in the soil profile. In the fallow, N_min_ was mostly in the deeper layers and had greater potential for leaching during fall and winter. However, in the SK treatment, N_min_ was in the top layers making it readily available for uptake by the subsequent crop. The N_min_ profile of the FK soil was intermediate. Organic N remained in the residue layer at the end of the experiment, but as mentioned before, we could not obtain a reliable measure of N concentration.

Even if N uptake by CC depleted the soil by April in the first season and seemed to increase the risk of pre-emptive competition with respect to the fallow, we propose that this may be a positive effect in many agricultural systems as it may help control N losses. In a tomato drip irrigation study, it was observed that high soil N_min_ levels at spring planting were linked to large leaching losses as crops are often overwatered to ensure establishment [49]. Excessive irrigation during the crop establishment period may represent up to 80% of total nitrate leaching losses [34], [50]. Therefore, keeping soil N_min_ at low levels and correcting fertilizer needs based on crop nutritional status during the cropping season holds promise for controlling N losses and increasing the efficiency of N use [51].

The larger above-ground biomass observed in the SK affected SWC. The CC extracted soil water by transpiration, increasing evapotranspiration losses when compared with the fallow [34]. Spring growth of the vetch/barley mixture was vigorous and soil water depletion was enhanced between the first and the second kill date. The differences in the whole profile SWC at the first kill date between CC treatments and the fallow were >35 mm in the first season and >55 in the second. At the second kill date, the SWC in the FK treatment was similar to the fallow. By the HPD and during the rest of the mulch period, the treatment effect on water availability was variable. While in the first season there was no difference, most likely because the soil was replenished by irrigation, the SWC was 60 mm lower in the SK than in the other treatments during the second season. Similar results were found in California [52], [53], where reductions in spring SWC were up to 80 mm due to CC extraction. In Minnesota, the difference in SWC in the upper 0.6 m caused by delaying the rye kill date by three weeks (from April to May) was 27 mm [12]. Our results confirm that late killing of CC increases pre-emptive water competition compared with early killing, a risk that might be mitigated in rainy years or by irrigation if water is available.

A relevant aspect of the increased water uptake by CC is the effect on the upper layers’ SWC. Sufficient top layer moisture at planting is crucial to ensure crop establishment and plant survival in many crops [49]. In the present study, the difference in SWC between the fallow and the CC treatments at the first kill date due to water depletion ranged between 13–19 mm in the upper 0.2 m of the soil and was enhanced between the fallow and the SK by the second kill date. However, the effect of CC residue mulching led to a higher SWC in the upper layers of the FK treatment than in the fallow at the HPD. When comparing CC treatments, the results are less clear; more water in the upper layers was retained in the SK than in the FK in the first season, but the SK was drier in the second. The FK treatment always increased water retention in the upper layers with respect to the fallow, but the result varied by year in the SK treatment. The top layer SWC was influenced by CC water uptake during winter, the residue mulch effect, and the amount of rain between the CC kill date and cash crop planting [9]. To clarify the discrepancy between the soil water depletion by CC uptake and conservation by the mulched CC residue, an experiment comparing sheltered and un-sheltered plots in Maryland showed that delaying kill date of a vetch CC produced more biomass and resulted in better soil moisture conservation in the upper 0.3 m [11]. However, recent experiments in Minnesota reported that delaying the rye kill date from the tillering to the booting stage came at the cost of SWC depletion in the top 0.3 m even if the residues continued to cover the soil after termination [12]. In Indiana, early kill of the CC resulted in better water conservation in the topsoil (0.1 m) than late kill. In drought years, early kill date produced an increase in maize yield but had a negative impact in normal precipitation years due to a lowering of soil temperature and workability at the maize planting date [7]. Our results indicate that early kill of the CC enhanced spring topsoil water moisture with respect to late kill, particularly by preserving soil water replenished by precipitation between kill dates.

A limitation found when comparing our study with others that focus on CC killing was that kill dates were always expressed as days after sowing. Given the variety of climatic regions in which cover cropping is practiced, the number of days after sowing is not a valid variable. We propose using degree-days accumulated and growth stage as more suitable variables for comparison between different regions. Soil type also has a major influence on the water balance and N cycling. Simulation models that can take multiple environmental factors into account might allow to overcome these limitations and increase the applicability of this and others studies to different locations and years.

**Figure 8:**
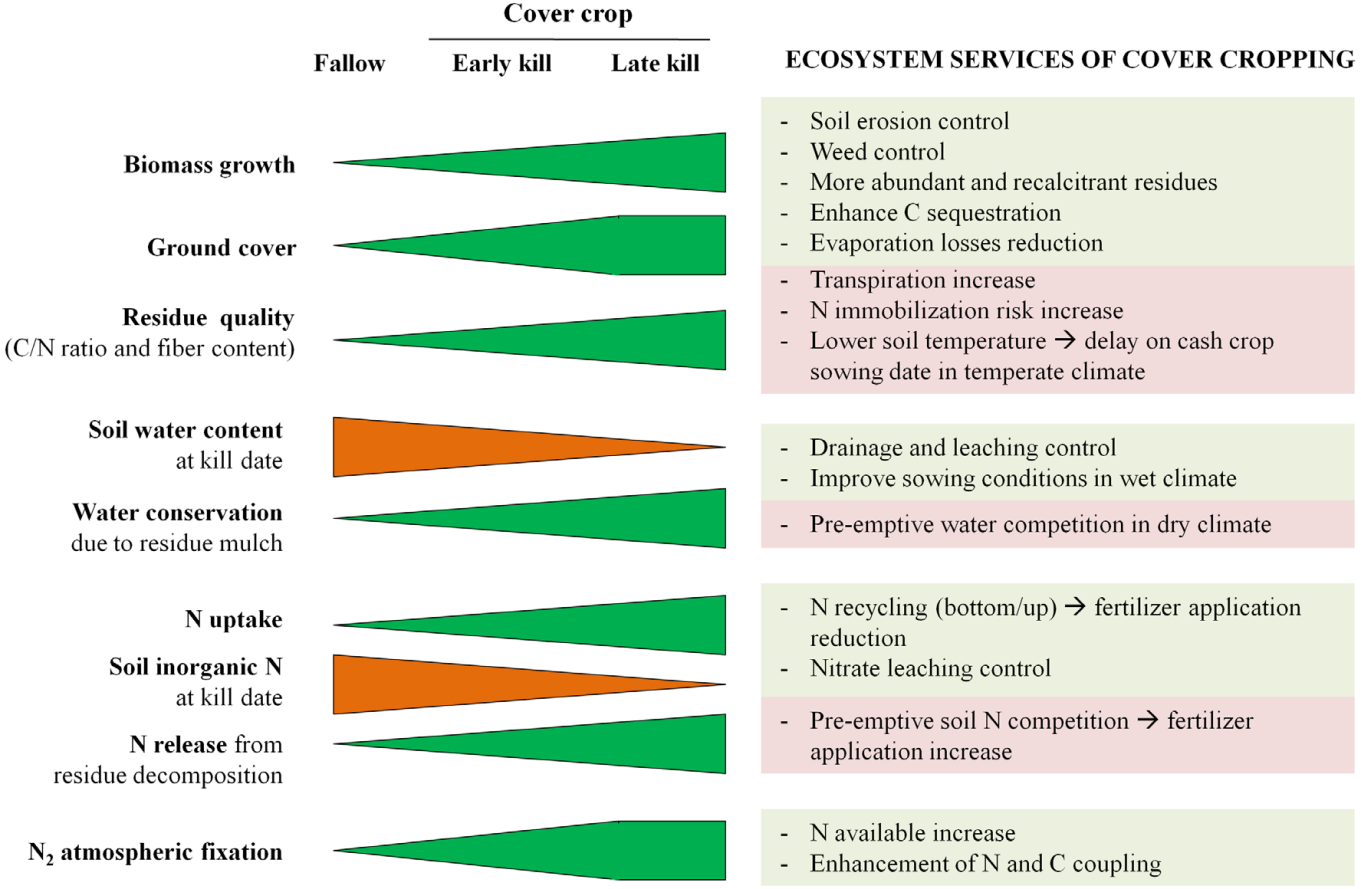
Schematic representation of the effect of kill date on environmental variables and ecosystem services with fallow as a reference condition.

One criticism of this study might be that a crop was not planted after the CC to obtain real data on crop water uptake. We are aware of this situation, but we think that this type of dataset is necessary to advance our understanding of the complex effect of CC kill date on crop resources. Separating the mulching and the effect of transpiration might allow for the calibration of key parameters, proper simulation of both processes, and finally, of the whole system. Replacing fallows by CC is known to provide many benefits, but the agronomic application needs to account for the requirements of specific agroecosystems. Kill date is a management tool to regulate the effects of CC on many environmental variables as presented in Figure 8. In dry environments, residue moisture conservation is the main goal of CC [9] whereas the risk of delaying sowing because of soils that are too wet is a major concern in cooler climates [7]. Depleting soil N enhances pre-emptive competition with the subsequent crop [6] but also controls N losses and creates opportunities for rational fertilizer application [34]. Late termination of CC maximizes their biomass production and the formation of a thick mulch, enhancing C sequestration, water conservation and weed control [36]. Our work does not close the debate on kill date but contributes to more rational decision-making for the successful use of CC.

## Conclusions

Kill date is a major management variable with CC that may lead to increasing water and N use efficiency and controlling N losses and pre-emptive competition with the subsequent crop. The ground cover of CC was >80% by the first kill date so no more benefits in terms of soil erosion or weed control derived from the living CC were expected by the second kill date. However, delaying kill date increased above-ground biomass, providing more CC residues with a higher fiber content and C/N ratio that were more recalcitrant to decomposition and, therefore, more suitable to protect the soil and enhance the slow release of the N pool.

The early kill date decreased the risk of pre-emptive competition by diminishing the N uptake of CC, and allowing more time for N release from CC residues. Cover crops generally uptake inorganic N from deeper layers to be released on the topsoil through residue mineralization. Delaying kill date enhanced this recycling effect that prevented losses of available N by keeping soil inorganic N at low levels. Kill date was a means of controlling this process and showed promise in mitigating N losses and increasing N use efficiency. The N mineralization potential in topsoil was enhanced by the presence of CC and by delaying kill date, proving that CC contribute to the enhancement of labile organic pools.

Delaying kill date increased pre-emptive competition of water, as the water extracted by CC from the first to the second kill date was greater than the water conservation due to the extra residue generated by the SK treatment. The soil water content in the upper layers at the time of planting the subsequent crop increased by the first kill date with respect to the fallow by preserving rain harvested between kill dates, but the soil water content in the late kill could be even lower than in the fallow depending on the climatic conditions of the year.

## Supporting Information

Table S1 **Biomass, N content and residue left.** Above-ground biomass and N content of cover crops at the first kill (FK) and second kill (SK) dates and residue covering the soil at the end of the mulch period (∼7 months after cover crop killing). Means with standard error in parentheses. Within a row, means with the same letter are not significantly different between kill dates at P<0.05.
(DOCX)

Table S2 **Chemical composition of cover crops.** Cover crop residue neutral detergent fiber (NDF), acid detergent fiber (ADF) and lignin (L) fractions and C/N ratio for the first kill (FK) and second kill (SK) dates. Means with standard error in parentheses. Within a row, means with the same letter are not significantly different between kill dates at P<0.05.
(DOCX)

Image S1 **Aerial view of the experimental site.** Plots are marked as fallow (FA), first kill (FK) nd second kill (SK) plus the replication number. Image taken between kill dates (March 29, 2012).
(TIF)

Image S2 **Ground level view of the experimental site.** Image taken between kill dates (April 4, 2012).
(TIF)
