# Supplementary material for: The Kill Date as a Management Tool for Cover Cropping Success
Source: PLoS One. 2014 Oct 8;9(10):e109587. doi: 10.1371/journal.pone.0109587 (PMC4190126; doi:10.1371/journal.pone.0109587)
Supplement: Table S1 — Biomass, N content and residue left. Above-ground biomass and N content of cover crops at the first kill (FK) and second kill (SK) dates and residue covering the soil at the end of the mulch period (∼7 months after cover crop killing). Means with standard error in parentheses. Within a row, means with the same letter are not significantly different between kill dates at P<0.05. (DOCX) [file pone.0109587.s001.docx]

|  | **Biomass (kg ha^-1^)** | |  | **N content (kg ha^-1^)** | |  | **Residue (kg ha^-1^)** | |
| --- | --- | --- | --- | --- | --- | --- | --- | --- |
|  | **2011-2012** | | | | | | | |
|  | FK | SK |  | FK | SK |  | FK | SK |
| Barley | 3390.4 (66.3) b | 5263.5 (580.6) a |  | 78.9 (6.4) b | 107.3 (11.5) a |  |  |  |
| Vetch | 1215.9 (158.2) | 1351.2 (377.3) |  | 45.4 (5.8) | 47.8 (13.2) |  |  |  |
| **Mixture** | **4606.4 (179.8) b** | **6614.6 (502.5) a** |  | **124.2 (10)** | **155.1 (12.9)** |  | **966.1 (175.4) b** | **2214.4 (304.6) a** |
|  |  |  |  |  |  |  |  |  |
|  | **2012-2013** | | | | | | | |
|  | FK | SK |  | FK | SK |  | FK | SK |
| Barley | 3623.3 (327.7) b | 5562.2 (494) a |  | 97.4 (4.3) b | 114.9 (1.7) a |  |  |  |
| Vetch | 2202.1 (138.8) | 2228.2 (133.6) |  | 89 (5) | 89.1 (5) |  |  |  |
| **Mixture** | **5825.5 (403.2) b** | **7790.3 (458.1) a** |  | **186.3 (6.8)** | **204 (6.2)** |  | **2151.5 (519.8) b** | **4014.0 (569.3) a** |
