# Supplementary material for: The Kill Date as a Management Tool for Cover Cropping Success
Source: PLoS One. 2014 Oct 8;9(10):e109587. doi: 10.1371/journal.pone.0109587 (PMC4190126; doi:10.1371/journal.pone.0109587)
Supplement: Table S2 — Chemical composition of cover crops. Cover crop residue neutral detergent fiber (NDF), acid detergent fiber (ADF) and lignin (L) fractions and C/N ratio for the first kill (FK) and second kill (SK) dates. Means with standard error in parentheses. Within a row, means with the same letter are not significantly different between kill dates at P<0.05. (DOCX) [file pone.0109587.s002.docx]

|  | **% NDF** | |  | **% ADF** | |  | **% L** | |  | **C/N** | |
| --- | --- | --- | --- | --- | --- | --- | --- | --- | --- | --- | --- |
|  | **2011-2012** | | | | | | | | | | |
|  | FK | SK |  | FK | SK |  | FK | SK |  | FK | SK |
| **Barley** | 45.5 (0.58) b | 56.35 (1.02) a |  | 17.3 (0.45) b | 22.02 (1.04) a |  | 1.22 (0.17) b | 2.91 (0.54) a |  | 18.1 (1.23) b | 21.2 (0.48) a |
| **Vetch** | 37.4 (1.1) b | 41.5 (0.74) a |  | 17.1 (0.97) b | 20.9 (0.73) a |  | 4.83 (0.48) | 5.91 (0.64) |  | 11.1 (0.16) b | 11.7 (0.17) a |
| **Mixture** | **43.4 (0.35) b** | **53.3 (1.62) a** |  | **17.3 (0.57) b** | **21.9 (0.8) a** |  | **2.19 (0.28) b** | **3.63 (0.22) a** |  | **16.2 (0.95) b** | **19.3 (0.78) a** |
|  |  |  |  |  |  |  |  |  |  |  |  |
|  | **2012-2013** | | | | | | | | | | |
|  | FK | SK |  | FK | SK |  | FK | SK |  | FK | SK |
| **Barley** | 50.9 (0.72) b | 59.1 (1.85) a |  | 24.6 (0.54) b | 29.9 (1.45) a |  | 1.69 (0.23) | 2.17 (0.23) |  | 15.1 (1.11) b | 20.1 (1.72) a |
| **Vetch** | 37.02 (0.39) b | 42.8 (1.23) a |  | 23.9 (0.53) b | 26 (0.5) a |  | 6.98 (0.31) | 6.59 (0.37) |  | 9.8 (0.09) b | 10.5 (0.22) a |
| **Mixture** | **45.6 (0.59) b** | **54.4 (1.09) a** |  | **24.3 (0.51) b** | **28.8 (0.95) a** |  | **3.69 (0.21)** | **3.45 (0.05)** |  | **13.1 (0.76) b** | **17.4 (1.34) a** |
