# Supplementary figures and images for: The Kill Date as a Management Tool for Cover Cropping Success
Source: PLoS One. 2014 Oct 8;9(10):e109587. doi: 10.1371/journal.pone.0109587 (PMC4190126; doi:10.1371/journal.pone.0109587)

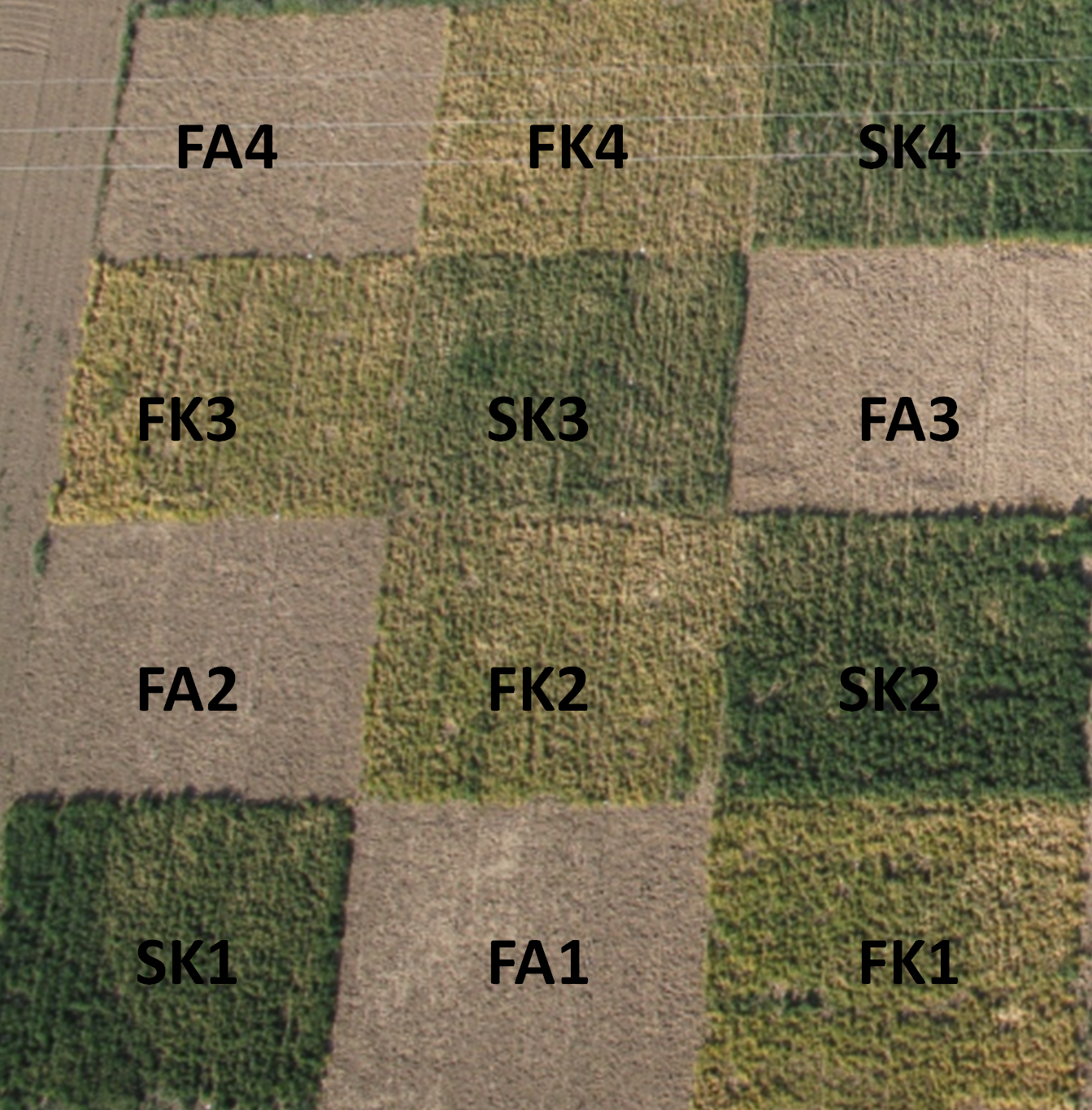

Supplement: Image S1 — Aerial view of the experimental site. Plots are marked as fallow (FA), first kill (FK) nd second kill (SK) plus the replication number. Image taken between kill dates (March 29, 2012). (TIF) [file pone.0109587.s003.tif]

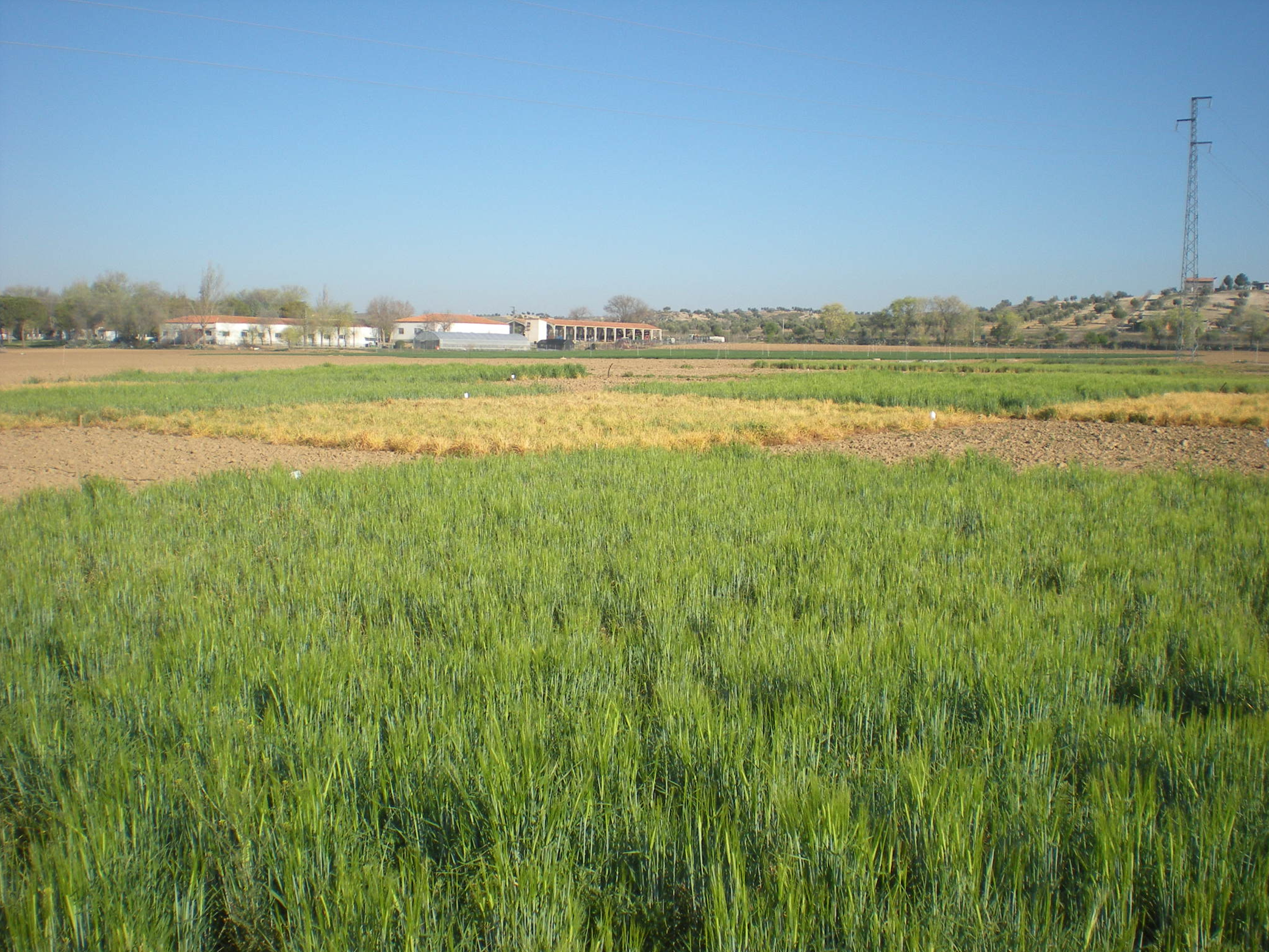

Supplement: Image S2 — Ground level view of the experimental site. Image taken between kill dates (April 4, 2012). (TIF) [file pone.0109587.s004.tif]
